# Evaluation of AZD1446 as a Therapeutic in DYT1 Dystonia

**DOI:** 10.3389/fnsys.2017.00043

**Published:** 2017-06-13

**Authors:** Chelsea N. Zimmerman, Karen L. Eskow Jaunarajs, Maria Meringolo, Francesca R. Rizzo, Massimo Santoro, David G. Standaert, Antonio Pisani

**Affiliations:** ^1^Center for Neurodegeneration and Experimental Therapeutics, Department of Neurology, University of Alabama-BirminghamBirmingham, AL, United States; ^2^Neurophysiology and Plasticity Laboratory, Fondazione Santa Lucia IRCCSRome, Italy; ^3^Department of Systems Medicine, University of Rome Tor VergataRome, Italy; ^4^Department of Neuroscience, Fondazione Don GnocchiMilan, Italy

**Keywords:** AZD1446, nicotine, DYT1, dystonia, cholinergic interneurons, dopamine, microdialysis, electrophysiology

## Abstract

DYT1 dystonia is an early-onset, hyperkinetic movement disorder caused by a deletion in the gene TOR1A, which encodes the protein torsinA. Several lines of evidence show that in animal models of DTY1 dystonia, there is impaired basal dopamine (DA) release and enhanced acetylcholine tone. Clinically, anticholinergic drugs are the most effective pharmacological treatment for DYT1 dystonia, but the currently used agents are non-selective muscarinic antagonists and associated with side effects. We used a DYT1 ∆GAG knock-in mouse model (DYT1 KI) to investigate whether nicotine and/or a non-desensitizing nicotinic agonist, AZD1446, would increase DA output in DYT1 dystonia. Using *in vivo* microdialysis, we found that DYT1 KI mice showed significantly increased DA output and greater sensitivity to nicotine compared to wild type (WT) littermate controls. In contrast, neither systemic injection (0.25–0.75 mg/kg) or intrastriatal infusion (30 μM–1 mM) of AZD1446 had a significant effect on DA efflux in WT or DYT1 KI mice. *In vitro*, we found that AZD1446 had no effect on the membrane properties of striatal spiny projection neurons (SPNs) and did not alter the spontaneous firing of ChI interneurons in either WT or DYT1 KI mice. We did observe that the firing frequency of dopaminergic neurons was significantly increased by AZD1446 (10 μM), an effect blocked by dihydro-beta-erythroidine (DHβE 3 μM), but the effect was similar in WT and DYT1 KI mice. Our results support the view that DYT1 models are associated with abnormal striatal cholinergic transmission, and that the DYT1 KI animals have enhanced sensitivity to nicotine. We found little effect of AZD1446 in this model, suggesting that other approaches to nicotinic modulation should be explored.

## Introduction

DYT1 dystonia is a form of early onset isolated dystonia (Albanese et al., 2016) stemming from a three base pair deletion (∆GAG) in the TOR1A gene in humans (Ozelius et al., 1997). Several genetic mouse models have been created to represent the disease and impaired basal release of dopamine (DA) in the striatum is a consistent feature (Balcioglu et al., 2007; Hewett et al., 2010). In particular, the DYT1 ∆GAG knock-in (DYT1 KI) mouse model shows reduced basal striatal DA levels and a blunted response to amphetamine-stimulated DA release compared to wild type (WT) littermates (Song et al., 2012). Genetic DYT1 rodent models also exhibit significant impairment of corticostriatal synaptic plasticity, a phenomenon that is directly linked to development of abnormal motor circuitry (Martella et al., 2009, 2014; Grundmann et al., 2012). Cholinergic function has been implicated in this phenotype since the loss of synaptic plasticity in genetic DYT1 mouse models can be rescued by anticholinergics (Maltese et al., 2014). Furthermore, the most effective therapy currently available for human DYT1 dystonia is the use of high doses of the non-selective muscarinic antagonist trihexyphenidyl, suggesting an important role for cholinergic transmission in the pathogenesis of dystonia.

While muscarinic antagonists improve dystonic symptoms, they come with a plethora of side effects (Jankovic, 2013) and patients could benefit from a different approach. The downstream actions of acetylcholine in the striatum are mediated by both metabotropic muscarinic receptors and ionotropic nicotinic receptors. Nicotine and nicotinic agents have long been studied in both Parkinson’s disease and levodopa-induced dyskinesia (Quik et al., 2007; Bordia et al., 2008; Huang et al., 2011), but they have yet to be explored for dystonia. Interestingly, smoking nicotine cigarettes has been reported to reduce symptoms in dystonic patients (Vaughan et al., 1997).

Nicotinic acetylcholine receptors (nAChR) are made up of pentameric assemblies of receptor subunits (α 2–8; β 2–4). They are broadly distributed across the brain. In the striatum, the most commonly expressed of the subunits are α4β2, which make up approximately 70% of striatal nAChR (Wada et al., 1989; Colquhoun and Patrick, 1997). These α4β2-containing nAChRs are located on both dopaminergic axons and non-dopaminergic neurons in the striatum, and play a major role in the modulation of DA release from nigrostriatal terminals (Chesselet, 1984; Giorguieff et al., 1977; Threlfell et al., 2012). For example, synchronous activation of striatal cholinergic interneurons (ChIs) can induce action-potential independent DA release via β2-containing nAChR found on nigrostriatal terminals (Cachope et al., 2012; Threlfell et al., 2012). In principle, enhancing striatal DA release through activation of nAChRs could help to alleviate symptoms associated with dystonia, without altering the activity of DA neurons themselves.

AZD1446 is a non-desensitizing α4β2 nicotinic acetylcholine receptor agonist developed from a series of N-acyldiazabicycle compounds (Mazurov et al., 2012). It has been investigated as a pharmacotherapeutic for neurological disorders, including attention deficit-hyperactivity disorder (ADHD) and levodopa-induced dyskinesia. In phase I clinical trials, AZD1446 was found to be generally safe and well tolerated in normal volunteers (Boström et al., 2014). It was also well-tolerated in both control and ADHD patients, though it did not display efficacy in treatment of ADHD symptoms (Jucaite et al., 2014). Studies in movement disorders have been limited, but a preliminary report described improvement in an animal model of levodopa-induced dyskinesia with AZD1446 administration (Mather, 2013). The advantage of employing a non-desensitizing agonist for nicotinic receptors is notable, since nAChRs are notorious for their fast desensitization in the presence of nicotine and other agonists (Quick and Lester, 2002).

We set out to first investigate the role of nicotine on striatal DA efflux in DYT1 KI mice and found that nicotine increases DA output in DYT1 KI compared to WT littermate controls. This result led us to believe that targeting nicotinic receptors and specifically, α4β2-containing nAChRs might be a viable therapeutic target for dystonia and might act through elevation of basal DA. We were also interested in the drug’s effects on ChIs and nigral neuronal firing, since previous research in the DYT1 KI mouse model has shown that there is increased basal acetylcholine and a decrease in basal DA levels in the striatum (Song et al., 2012; Thompson et al., 2015). In addition, ChIs have been shown to have abnormal properties in the DYT1 KI mouse model, with “paradoxical” excitation, rather than inhibition, in response to DA D2 agonists (Sciamanna et al., 2012; Martella et al., 2014). Due to the high number of nAChRs in the striatum and nicotine’s ability to increase DA in DYT1 KI mice, nicotine and nicotinic agonist could be potential therapeutic agents for dystonia patients. As such, the goal of the current set of experiments was to evaluate the effectiveness of AZD1446 on neurochemical and physiological measures associated with motor control in an established genetic mouse model of DYT1 dystonia.

## Materials and Methods

All experimental protocols were approved by the Institutional Animal Care and Use Committee at University of Alabama at Birmingham or the University of Rome “Tor Vergata”. Animal experiments were carried out in accord with NIH Guidelines, EC Internal Institutional Review Committee, EU directive or Italian rules (86/609/CEE, D.Lgs 116/1992, 2010/63EU, D.Lgs 26/2014), as appropriate. All members of the staff involved in the experiments have undergone a specific training for handling and working with rodents. All efforts were made to reduce the number of animals used and minimize their suffering.

### Animal Model

Heterozygous DYT1 mutant knock-in mice (Tor1A^∆E/+^; (Goodchild et al., 2005)) were maintained congenitally with C57BL/6J mice from Jackson Laboratories (Bar Harbor, ME, USA). Mice were housed with a 12 h light/dark cycle. Food and water were provided *ad libitum*. Tail DNA was genotyped using a primer pair to detect the 34 base pair loxP site in the DYT1 mutant (forward primer, AGTCTGTGGCTGGCTCTCCC; reverse primer, CCTCAGGCTGCTCACAACCAC). PCR products were run using a 2% agarose gel. Adult (3–6 months of age) male and female mutant (Tor1A^∆E/+^) and littermate controls (TorA^+/+^) were used for the following experiments.

### *In Vivo* Microdialysis

Male Tor1A^+/+^ and Tor1A^∆E/+^ littermates were anesthetized with isoflurane (1%–3%) and placed in a stereotaxic apparatus. Unilateral microdialysis cannulae (CMA Microdialysis, Stockholm, Sweden) were implanted vertically above the striatum (anterior +0.6; lateral +1.9; ventral −1.6 mm from bregma, according to the coordinates of Paxinos et al. (1985). Two anchor screws CMA Microdialysis) were implanted behind the cannula, which was then fixed to the skull with dental acrylic cement (Lang Dental, IL, USA). Following surgery, buprenorphine (0.03 mg/kg, i.p.) was injected for pain relief. Three to four days following surgery, the guide cannulae were removed and microdialysis probes were inserted (CMA7/2 mm, CMA Microdialysis). Mice were habituated to the microdialysis environment for 3 h while the probes were perfused with artificial cerebrospinal fluid (aCSF; 127.6 mM NaCl, 4.02 mM KCl, 750 μM NaH_2_PO_4_, 2.1 mM Na_2_HPO_4_, 2.00 mM MgCl_2_, 1.71 mM CaCl_2_; pH 7.4) at a constant rate of 2 μL/min for the duration of the experiment. Following habituation, samples were collected every 20 min. To determine the effect of nicotine on striatal DA release, following habituation, mice were striatally infused with 10 mM nicotine dissolved in aCSF for 1 h. To examine the effects of systemic AZD1446 on striatal DA, WT mice underwent a dose-response where AZD1446 was either systemically injected (0.25, 0.5, 0.75 mg/kg, ip) or striatally infused (30 μM, 300 μM, 1mM) for 1 h per dose. The genotype-specific effect of AZD1446 on striatal DA release was determined using reverse microdialysis (300 μM AZD1446) for 1 h in WT and DYT1 KI mice. Mice were killed following each procedure. To ensure correct placement of microdialysis probes, brains were post-fixed in 4% paraformaldehyde, cryoprotected in 30% sucrose after 48 h, and sectioned using a freezing microtome. Correct placement was visually determined to be 100% of all mice used.

### HPLC

For microdialysate collected during the nicotine infusion experiment, reverse-phase HPLC-ED was used to determine concentration of DA in each sample, according to the protocol of Kilpatrick et al. (1986), a method for semi-automated catecholamine and indoleamine analysis with coulometric detection. The system included an autoinjector (ESA, Model 542, Chelmsford, MA, USA), a solvent delivery system (ESA, Model 1582), and a MD-150 × 3.2 (150 × 3.2 mm, 3 μm packing) column (ESA). Samples were separated using a mobile phase composed of 90 mM sodium dihydrogen phosphate (monobasic, anhydrous), 0.05 mM EDTA, 1.7 mM octane sulfonic acid, and 9% acetonitrile, adjusted to pH 3.0 with o-phosphoric acid. Dialysate was analyzed for abundance of DA via an ESA coulometric detector (Coulochem II) coupled to a dual electrode analytical cell (ESA model 5014; first electrode at −100 mV, second electrode at +250 mV). The final oxidation current values were converted to nM concentration using a standard curve of known concentrations from 10^−6^ M to 10^−9^ M.

For the AZD infusion and systemic injection microdialysis experiments, DA was measured in microdialysate using HPLC-ED (Eicom HTEC-500 with Eicom Autosampler INSIGHT, San Diego, CA, USA) with an enzyme reactor (Eicom, AC-ENZYM II 4.6 × 30 mm) and an electrode (WE-3G; +400 mV vs. Ag/AgCl). The mobile phase consisted of a 50 mM dibasic potassium phosphate buffer (pH 8.5). Microdialysate sample chromatographs were analyzed based on established concentration curves for DA from 10^−6^ M to 10^−9^ M, and expressed as [DA] in nM.

### Slice Preparation

Preparation and maintenance of corticostriatal and midbrain slices were carried out as previously described (Mercuri et al., 1995; Sciamanna et al., 2011, 2012). Briefly, mice (6–8 weeks old) were deeply anesthetized and sacrificed by cervical dislocation. Brain was quickly removed and slices were cut using a vibratome in Krebs’ solution, continuously bubbled with 95% O_2_ and 5% CO_2_, containing (in mM): 126 NaCl, 2.5 KCl, 1.2 MgCl_2_, 1.2 NaH_2_PO_4_, 2.4 CaCl_2_, 11 glucose, 25 NaHCO_3_. Sagittal and coronal slices (270–300 μm for intracellular recordings; 200–210 μm for patch clamp recordings) were used for medium spiny projecting neurons (SPNs) and ChIs recordings, while horizontal slices (300 μm thickness) were used in order to better preserve substantia nigra. After 30–60 min recovery at room temperature, slices were transferred individually in the recording chamber (0.5–1 mL volume) and continuously superfused with oxygenated Krebs’ medium (2.5–3 mL/min at 32–33°C).

### Electrophysiology

A monochrome CCD Camera (Cohu 4912–3030, Poway, CA, USA) was used to visualize neurons on a PC monitor. Recordings were performed on individual cells by means of IR-DIC videomicroscopy with an Olympus BX51WI equipped with a 40× water immersion objective (XLUMPlan FL, Olympus). Intracellular recordings were performed blindly using sharp microelectrodes filled with 2 M KCl (40–60 MΩ). This electrophysiological approach was used to retain the fidelity of post-receptor signaling pathways. Glutamatergic excitatory postsynaptic potentials (EPSPs) were evoked with a bipolar electrode in the cortex (V–VI layer) in picrotoxin (50 μM) to block GABA receptors. Synaptic stimuli were delivered at 0.1 Hz, and six events were averaged. Paired-pulse facilitation was assessed by presenting two stimuli, which evoked synaptic responses at ~50% of maximal amplitude, with an interstimulus interval of 50 ms and measuring the ratio of the peak amplitude of the second EPSP (EPSP2).

Patch clamp recordings, either in cell-attached or whole-cell configuration, were made using borosilicate glass pipettes (1.5 mm outer diameter, 0.86 mm inner diameter) pulled on a P-97 Puller (Sutter Instruments, CA, USA). Pipettes resistances ranged from 3 MΩ to 5 MΩ. Membrane currents were continuously monitored and access resistance, measured in voltage-clamp, was in the range of 5–30 MΩ. For cell-attached or whole-cell recordings on ChIs, pipettes were filled with K^+^ internal solution containing (in mM): 125 potassium gluconate, 10 NaCl, 1.0 CaCl_2_, 2.0 MgCl_2_, 0.1 1,2-bis(2-aminophenoxy)ethane-N,N,N,N-tetra-acetic acid (BAPTA), 10 HEPES, 0.3 GTP, 2.0 MgATP; pH adjusted to 7.3 with KOH. Under voltage-clamp conditions, cells were clamped at −60 mV and series resistance (8–15 MΩ) was monitored during the course of the experiment by the peak amplitude of the capacitive transient induced by a −5 mV pulse. Signal acquisition and offline analysis were performed with an Axoclamp 2B (Axon Instruments) and pClamp9 software (Molecular Devices, Sunnyvale, CA, USA).

Extracellular single-unit signals were recorded at 34 ± 0.5°C from dopaminergic neurons of the substantia nigra pars compacta (SNpc) located in a submerged chamber with aCSF flowing at 2.5–3 mL/min, on the stage of an upright microscope (Axoscope FS, Zeiss, Gottingen, Germany), equipped for infrared video microscopy (Hamamatsu, Tokyo). Glass microelectrodes had a tip resistance of 10–20 MΩ and were filled with aCSF. The recordings were performed with an Axoclamp 900A amplifier (Axon Instruments, Foster City, CA, USA), using Clampex 10 software and digitized with a Digidata 1440A. Dopaminergic neurons, selected on the basis of their localization and morphology, were identified by their well-known properties, such as the presence of a regular spontaneous firing (0.5–4 Hz), firing inhibition in response to DA (Grace and Onn, 1989; Mercuri et al., 1995). Changes in firing rate are expressed as normalized to the basal firing rate (mean firing rate during 3 min prior to drug application) and were measured after drug perfusion.

### Data Analysis and Statistics

Electrophysiological data were analyzed using pClampfit 10.5 (Molecular Device) and Prism 6.0 e (GraphPad). Numerical data are presented as means ± SEM; n values represent independent observations. The evaluation of statistical difference was performed using Student’s *t*-tests. Microdialysis data were analyzed using a one-way ANOVA for the AZD1446 microdialysis dose response, and one-sample *t*-tests were used to determine significance of percent baseline DA induced by AZD1446 and nicotine in WT and DYT1 KI mice. The significance level was set at *p* < 0.05.

### Drug Source and Handling

(-)Nicotine hydrogen tartrate salt, DA hydrochloride and CNQX were obtained from Sigma-Aldrich (St. Louis, MO, USA), whereas picrotoxin and MK-801 were obtained from Tocris (UK). AZD1446 was obtained from AstraZeneca (Sweden). For electrophysiological experiments, drugs were prepared in stock solutions and bath-applied at known concentrations via a three-way tap system. A complete exchange of the solution in the recording chamber occurred in about 1 min. For microdialysis, nicotine was dissolved in aCSF and pH adjusted to 7.4. AZD1446 was prepared in stock solution in aCSF for striatal infusion and in water for systemic injections.

## Results

### DYT1 KI Mice have Increased Sensitivity to Nicotine Compared to WT Mice

**Figure 1:**
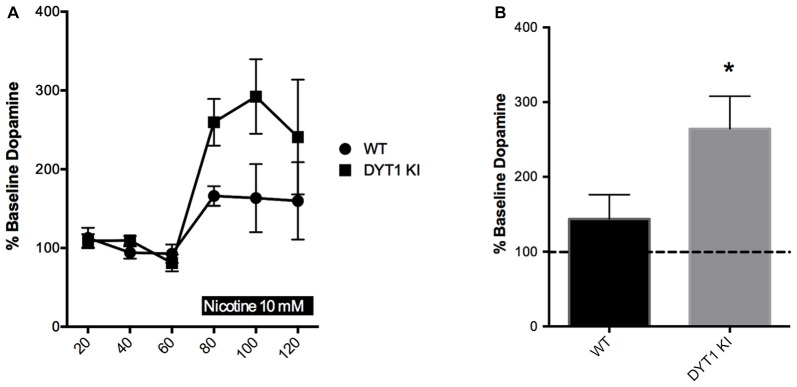
Nicotine increases dopamine (DA) efflux in DYT1 KI mice compared to littermate controls in *in vivo* microdialysis. **(A)** Time course of each sample taken **(B)** DYT1 KI mice show significantly increased DA efflux in response to nicotine (10 nM) compared to WT littermate controls; *p < 0.05.

Previous physiological studies have indicated that nicotinic receptor activation by tonic acetylcholine release is needed to ensure that striatal DA terminals are sensitive to stimulation (Rice and Cragg, 2004). In addition, nicotinic receptors may independently modulate striatal DA release through synchronized activity of ChIs (Threlfell et al., 2012). Based on previous data indicating that there is a hypercholinergic state present in DYT1 dystonia (Thompson et al., 2015), we expected that high levels of acetylcholine in DYT1 animals might render DA terminals more sensitive to nicotine’s DA-stimulating effects. Basal levels of DA were not different between WT and DYT1 KI mice (0.277 ± 0.0862 and 0.545 ± 0.3380 nM, respectively). We found that over the 60 min treatment period, the WT mice did not show a significant change in extracellular DA compared to baseline in response to nicotine. In contrast, DYT1 KI mice showed a marked increase in nicotine-induced DA release compared to baseline (Figures 1A,B unpaired *t*-test; ANOVA *p* < 0.05).

### AZD1446 Does Not Alter Striatal DA Release in a Mouse Model of DYT1 Dystonia

**Figure 2:**
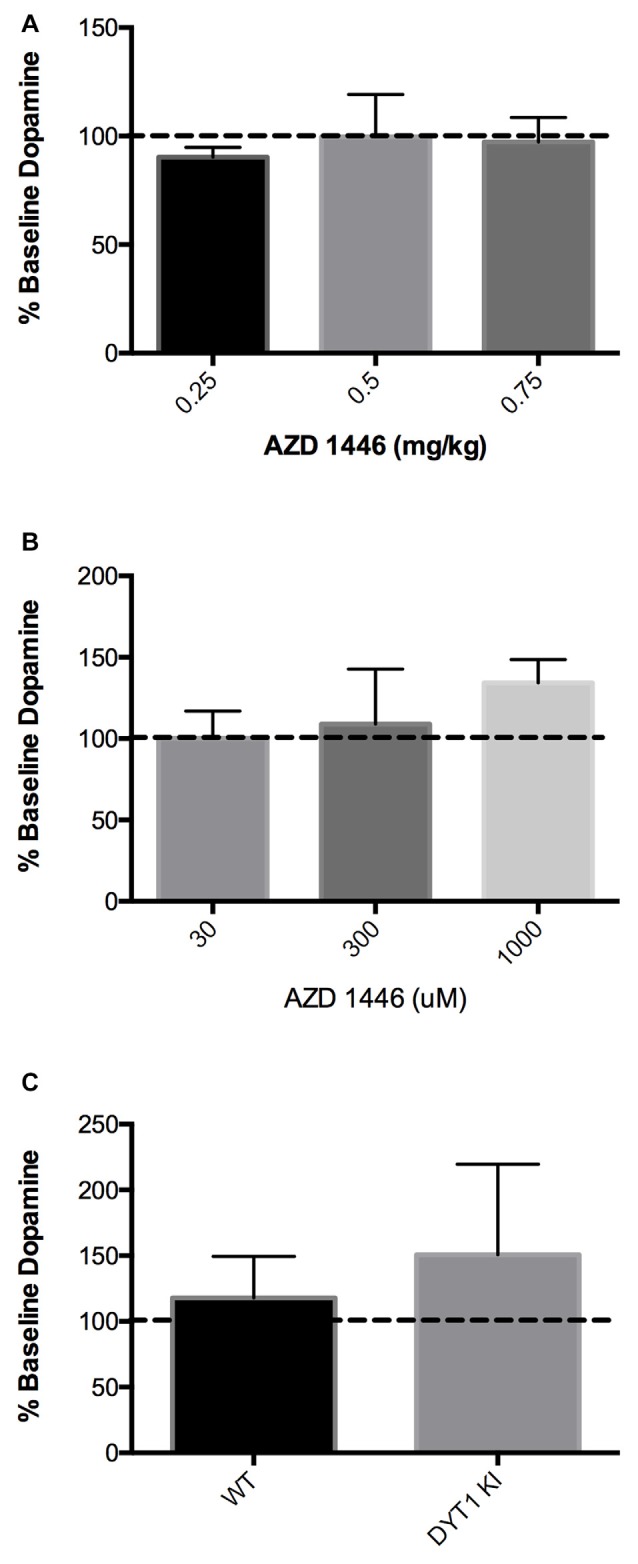
AZD1446 has no effect on striatal DA efflux via *in vivo* microdialysis **(A)** WT mice showed no significant increase of striatal DA when AZD1446 was systemically injected at 0.25 mg/kg, 0.5 mg/kg and 0.75 mg/kg (ip). **(B)** WT mice showed no significant increase in striatal DA when AZD1446 was striatally infused at 30 μM, 300 μM or 1 mM. **(C)** Neither WT or DYT1 KI mice showed a significant increase in DA efflux with 300 μM AZD1446 added to artificial cerebrospinal fluid (aCSF).

Since DYT1 KI mice showed an increase in striatal DA in response to nicotine, we explored whether AZD1446 could enhance striatal DA efflux when delivered either systemically or intrastriatally in a DYT1 knock-in mouse model. When AZD1446 was given systemically in WT mice, no difference in extracellular DA was observed in doses ranging from 0.25 mg/kg to 0.75 mg/kg (Figure 2A; WT (*n* = 4), DYT1 KI (*n* = 4); ANOVA, *p* > 0.05). Since there was no difference seen systemically, we explored local intrastriatal administration of AZD1446 by reverse microdialysis. In WT mice, AZD1446 did not affect extracellular striatal DA across a range of doses from 30 μM to 1000 μM (Figure 2B; WT (*n* = 4), DYT1 KI (*n* = 5); ANOVA, *p* > 0.05). Based on these data and in order to allow for a bidirectional change in DA output in DYT1 KI mice, a 300 μM concentration of AZD1446 was employed to determine if the DYT1 mutation would alter the response of striatal DA efflux to this drug. However, AZD1446 failed to elicit a significant increase in DA output in either WT or DYT1 KI mice (Figure 2C; unpaired *t*-test, *p* > 0.05).

### Electrophysiological Effects of AZD1446 on SPNs and ChIs

SPNs and ChIs were recorded from dorsal striatum and identified on the basis of their morphology and peculiar electrophysiological features. Collectively, basic membrane properties of SPNs and ChIs measured from WT and DYT1 KI slices showed no differences between the two genotypes, as previously described (Martella et al., 2014).

**Figure 3:**
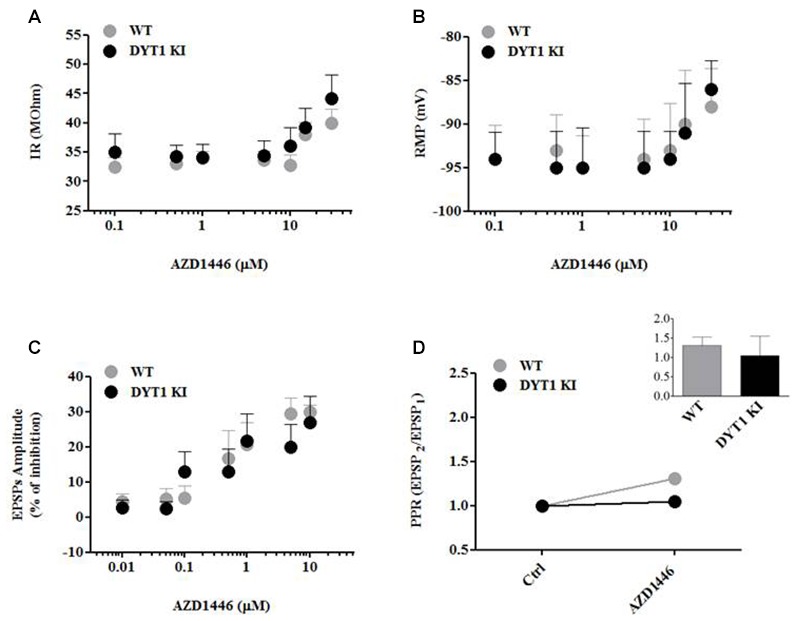
AZD1446 effects on intrinsic membrane and synaptic properties of spiny projection striatal neurons (SPNs), and paired pulse ratio (PPR). **(A,B)** Dose-response curves of input resistance (IR) and resting membrane potential (RMP) after bath application of increasing concentrations of AZD1446 (0.1–30 μM). Note, at higher concentrations, a slight depolarization and an increase of membrane resistance of recorded neurons. **(C)** Dose-response curve of excitatory postsynaptic potentials (EPSPs) amplitude (expressed as % of inhibition) measured at increasing doses of AZD1446 (0.01–10 μM). AZD1446 dose-dependently inhibits corticostriatal EPSP in both genotypes. Each data point represents the mean of at least five independent observations. **(D)** Paired pulse facilitation (50 ms interstimulus interval). Plot does not show significant difference between WT and DYT1 KI mice after bath application of AZD1446 (1 μM; ANOVA *p* > 0.05).

In order to characterize the mechanism of action of AZD1446, we performed intracellular recordings on SPNs before and after acute bath-application of known drug concentrations. Low and moderate concentrations of AZD1446 (0.01–5 μM) failed to modify intrinsic properties of SPNs recorded from WT and DYT1 KI mice; in contrast, high concentrations (>5 μM) of AZD1446 produced a significant depolarization linked with an increased membrane resistance (Figures 3A,B). Then, we investigated the effect of AZD1446 on the amplitude of corticostriatal EPSPs. We observed a dose-dependent inhibitory effect without any significant differences between genotypes, with a maximal inhibition, respectively, of 30.0 ± 2.0% (WT) and 27.0 ± 7.36% (DYT1 KI) at the maximal dose tested (10 μM; Figure 3C). To evaluate the effect of AZD1446 on short-term plasticity we measured the paired pulse ratio (PPR). No significant differences in the PPR were found between WT and DYT1 KI mice (Figure 3D; WT (*n* = 18) 1.308 ± 0.22%; DYT1 KI (*n* = 23) 1.05 ± 0.5%; ANOVA *p* > 0.05).

**Figure 4:**
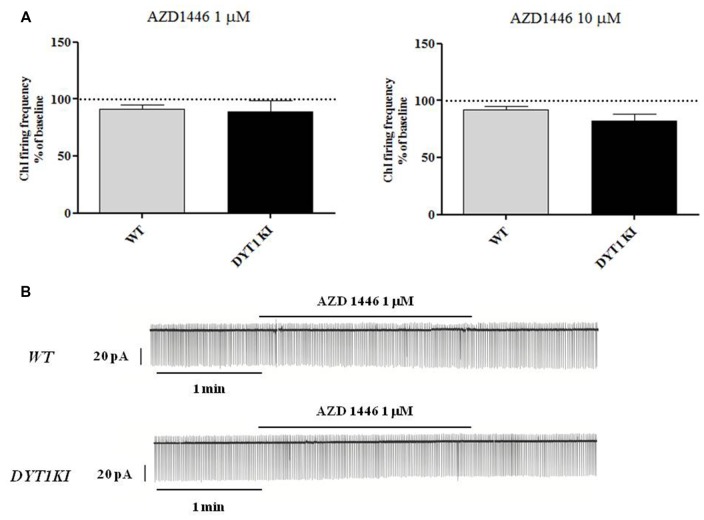
AZD1446 does not produce statistically significant changes on the firing frequency of striatal cholinergic interneurons (ChIs). **(A)** Plots show firing frequency of ChIs (expressed as % of baseline) after bath-perfusion of two different doses of AZD1446 (1 μM and 10 μmM, 2 min). No significant differences were found in two genotypes at two tested doses (ANOVA *p* > 0.05). **(B)** Representative traces of spontaneous firing activity of ChIs recorded in a cell-attached patch clamp configuration, before and after bath application of AZD1446 (1 μM, 2 min).

**Figure 5:**
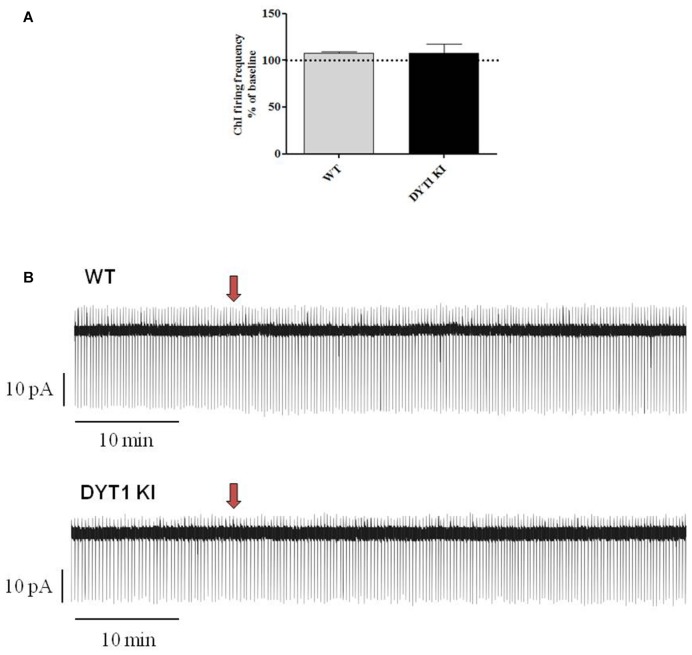
Effect of a single fast perfusion application of AZD1446 on firing frequency of striatal ChIs. **(A)** Summary bar chart of the effect of a single delivered puff of AZD1446 (1 μM, 500 ms). AZD1446 does not produce any significant effect on firing frequency of ChIs (ANOVA *p* > 0.05). **(B)** Sample traces showing the firing rate recorded after fast application of AZD1446 (1 μM, 500 ms), respectively in WT and DYT1 KI mice. Red arrow shows the release of puff.

ChIs are known to play a key role in regulating DA release (Rice et al., 2011; Cachope et al., 2012; Kossillo et al., 2016). In striatal slices, cholinergic activity acts locally and potently regulates DA release (Zhou et al., 2001). Previous patch-clamp studies in slices have revealed that nicotine (100 μM) increases action potential frequency in striatal ChIs (Matsubayashi et al., 2001). Bath-application of two different doses of AZD1446 (1 μM and 10 μM, 2 min) did not produce significant effects on resting membrane potential (RMP) or spontaneous firing activity of the recorded striatal ChIs (Figure 4; 1 μM: WT (*n* = 3) 91.2 ± 3.02%; DYT1 KI (*n* = 6) 89.2 ± 9.66%; 10 μM: WT (*n* = 4) 92.2 ± 2.95%; DYT1 KI (*n* = 4) 91.2 ± 7.30%; ANOVA, *p* > 0.05). To rule out a possible desensitizing effect, AZD1446 was delivered by means of a single puff, allowing a focal, fast drug perfusion. AZD1446 (1 μM, 500 ms) did not produce a significant effect on firing frequency or membrane potential of ChIs, both in WT and DYT1 KI mice (Figure 5; WT (*n* = 4) 107.3 ± 2.11%; DYT1 KI (*n* = 6), 108.1 ± 9.38%; *p* > 0.05).

### AZD1446 Has a Small but Significant Effect on Nigral Firing

**Figure 6:**
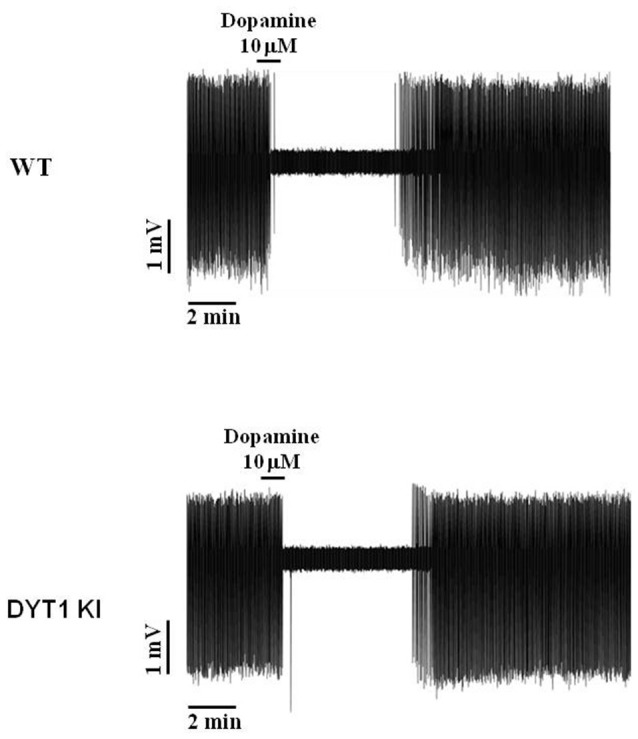
Representative traces of dopaminergic neuron spontaneous firing inhibition by bath perfusion of exogenous DA. Transient inhibitory response to application of exogenous DA (10 μM, 1 min) in WT and DYT1 KI dopaminergic neurons, with a complete wash-out of firing frequency in about 6–8 min.

Since no effect was seen on striatal ChIs, we looked directly at dopaminergic neuron firing in the substantia nigra to determine if AZD1446 could increase nigral cell firing. Dopaminergic cells are spontaneously active *in vitro*, firing action potentials in a slow, regular pattern (Grace and Onn, 1989; Lacey et al., 1989). Accordingly, during extracellular recordings we observed a characteristic pacemaking activity pattern in both WT and DYT1 KI neurons, and a transient inhibitory effect during perfusion of exogenous DA (DA, 10 μM, 1 min) with a complete washout in about 6–8 min (Figure 6). To study the effect of AZD1446 on the spontaneous firing of dopaminergic neurons, we used extracellular recordings that do not perturb the cytoplasmic content.

**Figure 7:**
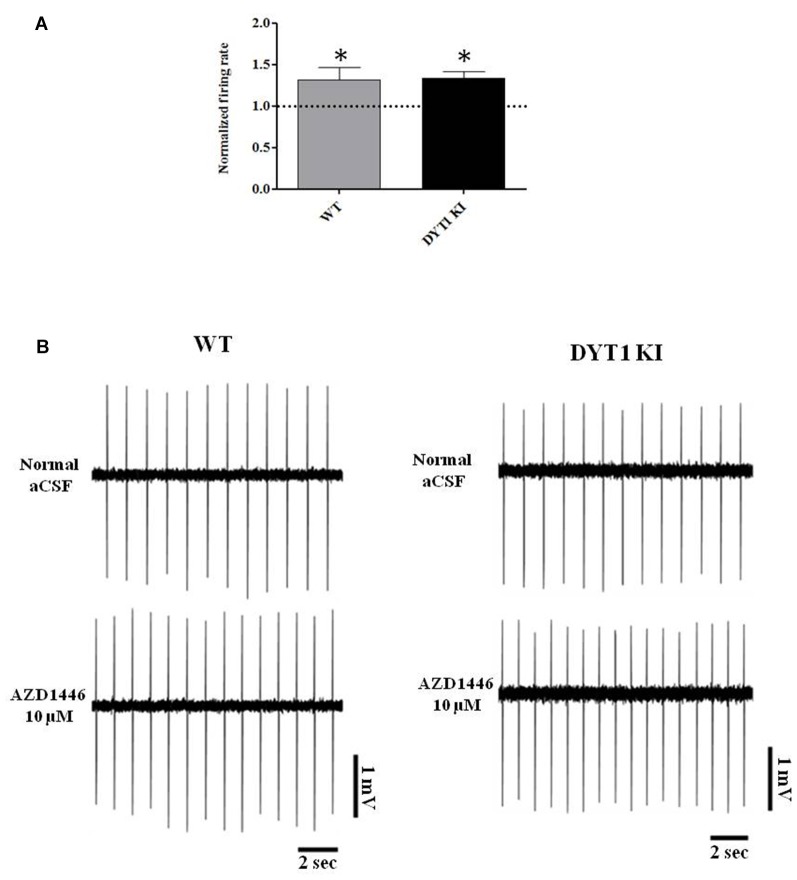
AZD1446 increases the firing rate of nigral dopaminergic cells. **(A)** Plot shows normalized firing frequency of dopaminergic neurons after bath perfusion of AZD1446 (10 μM, 4 min). Note the increase of firing rate in both genotypes (*t*-test paired *p < 0.05). **(B)** Representative traces of firing frequency of nigral cells in control condition (above) and after bath perfusion of AZD1446 (below).

**Figure 8:**
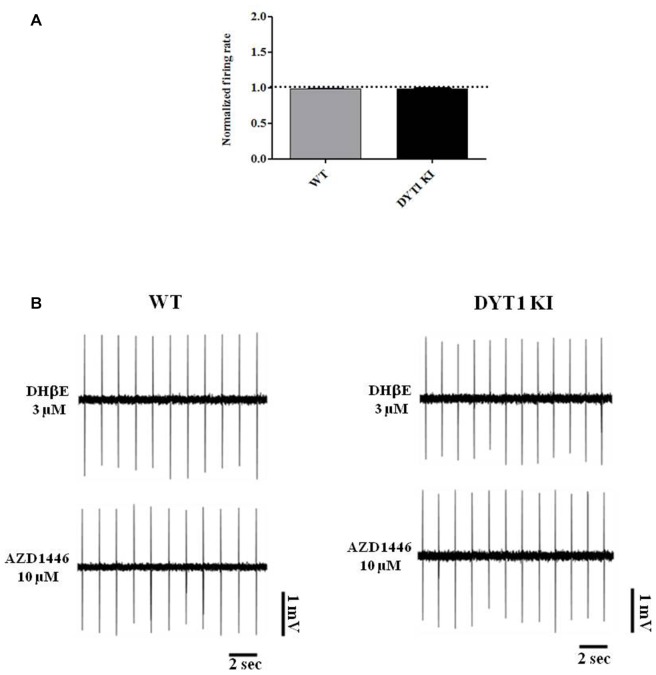
α_4_β_2_ nicotinic receptor antagonist dihydro-beta-erythroidine (DHβE) blocks AZD1446 effect on nigral cells recording. **(A)** Plot of normalized firing rate of nigral dopaminergic neurons. Pre-incubation of α_4_β_2_ nicotinic receptor antagonist DHβE (3 μM) blocks the effect of AZD1446 (10 μM, 4 min), (*t*-test unpaired *p* > 0.05). **(B)** Representative traces of firing frequency after pre-incubation of DHβE (above) and after added AZD1446 (below).

Perfusion with AZD1446 (10 μM, 4 min) caused a modest but significant increase of spontaneous firing activity of recorded cells both in DYT1 KI and WT slices (Figure 7; WT (*n* = 5): 1.321 ± 0.1139, paired *t*-test *p* = 0.048; DYT1 KI (*n* = 4): 1.336 ± 0.07; paired *t*-test *p* = 0.0224). No difference was found by comparing the two strains (Figure 7; unpaired *t*-test, *p* = 0.9264). In accord with the pharmacological profile of AZD1446, pretreatment with the selective α4β2 receptor antagonist DHβE (3 μM, 10 min) prevented the excitatory effect of AZD1446 on nigral neurons in both animal groups (Figure 8; WT 0.99 ± 0.01; DYT1 KI 0.98 ± 0.01; unpaired *t*-test, *p* < 0.05; *n* = 4).

## Discussion

The goal of this study was to determine the effect of AZD1446 on striatal function in an animal model of DYT1 dystonia. In this study, we found that DYT1 KI mice are more sensitive than WT animals to nicotine’s DA stimulating effects when the drug is applied directly to the striatum *in vivo*. However, the α4β2-specific, non-desensitizing nAChR agonist AZD1446 did not have significant effects on DA efflux systemically or intrastriatally in either WT or DYT1 KI mice. *In vitro*, AZD1446 did not alter membrane properties or firing of ChIs in DYT1 KI mice. While there was no effect on ChIs, AZD1446 had a small effect on dopaminergic neuron firing which could be blocked with nicotinic antagonist DHβE.

Nicotinic receptors have long been known to regulate DA release. Each subunit of the pentameric structure has different properties and contributes to DA efflux differently. α7 nAChR subunits are usually found in a homomeric state and mostly found on dopaminergic neuron terminals (Quick and Lester, 2002). In the striatum, α4β2 is the most abundant subunit composition (Fenster et al., 1999) and is mostly found on the dopaminergic cell bodies and on GABA neurons in the striatum. Nicotine itself has complex actions because it can both activate and desensitize nicotinic receptors (Quick and Lester, 2002). Through *in vivo* microdialysis, chronic nicotine has been shown to significantly increase DA efflux in the striatum of WT mice (Salas and De Biasi, 2008).

Basic physiological studies indicate that tonic acetylcholine release acts on nicotinic receptors on striatal dopaminergic terminals, rendering them sensitive to action potentials (Rice and Cragg, 2004). We expected that the hypercholinergic state of the striatum found in rodent models of DYT1 dystonia might enhance nicotinic receptor sensitivity. Indeed, we found that DYT1 KI animals were more sensitive to intrastriatal delivery of nicotine (Figure 1). The mechanisms of this enhanced sensitivity may relate to changes in receptor sensitivity downstream of the hypercholinergic state. Chronic nicotine exposure is known to upregulate a type of α4β2-containing nicotinic receptor that is highly sensitive to cholinergic stimulation (Buisson and Bertrand, 2001; Nelson et al., 2003; Kuryatov et al., 2005; Sallette et al., 2005; Vallejo et al., 2005). It is possible that the effect of the hypercholinergic striatal environment in DYT1 animals is similar, and contributes to the enhanced sensitivity to nicotine’s action on DA outflow in DYT1 KI mice shown here.

AZD1446 is a non-desensitizing nicotinic agonist selectively acting on α4β2-containing nAChRs. It is of particular interest because it has been used in human subjects, where it has been found to be generally safe and well tolerated (Mazurov et al., 2012). Thus, it is a reasonable candidate to explore in the context of dystonia. Despite the logic of this approach, in the current study we found that AZD1446, across a wide range of doses, failed to alter striatal DA efflux when given either intrastriatally or systemically (Figure 2). Further, *in vitro* there was no effect on ChI firing activity in WT or DYT1 KI mice (Figures 3, 4). We did observe an inhibitory effect on corticostriatal EPSP amplitude, but the functional significance of these effects is uncertain at best, especially considering the distribution of α4β2 subunits in the striatum. Conversely, a small increase in dopaminergic neuron firing in response to AZD1446 was measured, consistent with the previously described excitatory effect of nicotine on these cells, and in line with presence of α4β2 postsynaptic nAChRs on nigral neurons (Clarke et al., 1987; Matsubayashi et al., 2004).

Collectively, our data indicate that AZD1446 is not a particularly promising candidate for development as a treatment for DYT1 dystonia. Nicotinic agents as a class, however, do appear to have substantial promise, as illustrated by the differences in DA release in response to nicotine in DYT1 and WT animals. As such, nicotinic agents likely have a place in the field of DYT1 dystonia and their effects should continue to be studied to help understand more about the disease and possible therapeutics.

## Author Contributions

DGS, AP and CNZ designed the research; MM, FRR, MS and CNZ performed the research; MM, FRR and CNZ analyzed the data; CNZ, KLEJ, DGS and AP wrote the manuscript; DGS and AP obtained funding.

## Conflict of Interest Statement

The authors declare that the research was conducted in the absence of any commercial or financial relationships that could be construed as a potential conflict of interest.
